# A Muscle-Based Approach to Superior Orbital Sulcus Hollowness: Preseptal Orbicularis Oculi Flap in Upper Blepharoplasty

**DOI:** 10.1007/s00266-026-06020-w

**Published:** 2026-06-22

**Authors:** Mustafa Akyurek, Buruc Barkin Battal, Gökay Karaemir, Orhun Ali

**Affiliations:** Department of Plastic, Reconstructive and Aesthetic Surgery, Faculty of Medicine, Çanakkale Onsekiz Mart University, 17100 Canakkale, Turkey

**Keywords:** Upper blepharoplasty, Superior orbital sulcus hollowness, Orbicularis oculi muscle flap, FACE-Q

## Abstract

**Background:**

Superior orbital sulcus hollowness is a common aesthetic concern that may result from structural changes, trauma, or excessive fat removal during upper blepharoplasty, and it can be further exacerbated by age-related orbital remodeling. Traditional corrective approaches such as fat redistribution and autologous fat grafting carry limitations, including graft atrophy and the risk of embolic complications. This study evaluated the safety and outcomes of superior orbital sulcus correction using a medial-pedicled preseptal orbicularis oculi muscle flap.

**Methods:**

This retrospective study analyzed 481 patients who underwent upper blepharoplasty between January 2017 and June 2024, of whom 45 received additional correction of superior orbital sulcus hollowness with a medial-pedicled preseptal orbicularis oculi muscle flap. Exclusion criteria included male sex, previous upper eyelid surgery, levator dehiscence, less than six months of follow-up, and refusal to complete the FACE-Q Adverse Effects questionnaire. The mean follow-up duration was 8 months, and the mean patient age was 51.4 years. Postoperative complications and patient-reported outcomes were evaluated and compared with those of patients undergoing conventional upper blepharoplasty.

**Results:**

Early postoperative complications, including transient lagophthalmos and edema, were self-limiting. In the conventional blepharoplasty group, 16 patients developed medial canthal scarring, with four requiring revision. In the flap group, transient supraorbital hypoesthesia occurred in 71% of patients and resolved within a few months, and no cases of flap necrosis were observed. Statistical analysis using the Mann–Whitney U test demonstrated no significant difference in FACE-Q scores between the two groups (U = 8828.0, p = 0.251).

**Conclusion:**

The medial-pedicled preseptal orbicularis oculi muscle flap appears to be a safe, reproducible, and anatomically sound technique for selected patients with superior orbital sulcus hollowness. Although the relatively uniform flap volume may not fully correct the deformity in all cases and patient numbers were limited, this method provides a promising alternative to fat grafting without adding long-term complications, with the potential to enhance aesthetic outcomes and patient satisfaction.

*Level of Evidence IV* This journal requires that authors assign a level of evidence to each article. For a full description of these Evidence-Based Medicine ratings, please refer to the Table of Contents or the online Instructions to Authors www.springer.com/00266.

**Supplementary Information:**

The online version contains supplementary material available at 10.1007/s00266-026-06020-w.

## Introduction

The eyelid is one of the most critical facial locations from an aesthetic point of view, and the young and vibrant look is the center of an aesthetic face [1]. Upper blepharoplasty remains the most common procedure for rejuvenating the upper eyelid, and the surgical techniques have also been clearly demarcated in the literature, largely based on the surgeon’s experience and preference [2]. The most frequent approach involves resection of the skin and strip resection of orbicularis oculi muscle for a sufficient supratarsal fold. Notably, studies have established that removal of the orbicularis oculi muscle does not alter the eyelid pattern, thus, relying on the surgeon’s preference to remove it [3, 4].

Aging process creates significant changes in the aesthetic as well as the functional aspect of the human face. Particularly, the remodeling of bony structures in the orbital area with the passage of time significantly impacts the shape and appearance of the periorbital region. Over time, some individuals may experience a loss of orbital support and develop hollowness or deepening of the superior orbital sulcus, resulting in an aesthetically unpleasing look, regardless of surgical history [5, 6].

This study aims to address the aesthetic concerns of patients with age-related loss of orbital support or those who develop medial superior orbital sulcus hollowness [7]. We propose the use of a repositionable and moldable muscle flap harvested from the preseptal portion of the ipsilateral orbicularis oculi muscle to restore volume and achieve a more natural and aesthetically pleasing upper eyelid contour. The preseptal orbicularis oculi muscle flap (POOM) represents an innovative technique, allowing for customized augmentation based on individual anatomy and offering the potential for improved outcomes in the correction of superior orbital sulcus hollowness associated with aging.

## Materials and Methods

This retrospective study included all patients who underwent upper blepharoplasty at our institution between January 2017 and June 2024. The study was approved by the local institutional review board and conducted in accordance with the Declaration of Helsinki.

All patients who underwent upper blepharoplasty during the study period were identified from surgical records. Patients were divided into two groups based on the surgical technique performed: those who underwent conventional upper blepharoplasty alone (Group 1), and those presenting with superior orbital sulcus hollowness who underwent correction with a POOM flap in addition to blepharoplasty (Group 2).

Exclusion criteria were as follows: male patients, patients with a history of previous upper eyelid surgery, patient with levator dehiscence, those with less than six months of postoperative follow-up, and patients who declined to complete the FACE-Q Adverse Effects questionnaire [8]. For all included patients, demographic data (age, sex), surgical details, perioperative complications, and standardized preoperative and postoperative photographs were collected. Patient complaints and complications documented at each follow-up visit were reviewed. All patients were asked to complete the FACE-Q Adverse Effects module at the 6-month postoperative follow-up. The primary outcome measure was the comparison of FACE-Q Adverse Effects scores between the two groups. Statistical analysis was performed to compare demographic characteristics and outcomes. Continuous variables are presented as mean ± standard deviation or median (interquartile range), as appropriate. Categorical variables are reported as frequencies and percentages.

### Surgical Technique

The patient was placed in a seated position to facilitate accurate incision markings, extending from the supratarsal fold to the lateral border of the orbicularis oculi muscle, aligning with existing crow’s feet lines whenever feasible. The cranial boundary of the skin excision was determined by pinching the skin at the lateral canthus level with an Adson forceps, marking the peak point of the cranial incision line. For patients presenting with superior orbital sulcus hollowness, the deepest point of the deformity was marked. Local anesthesia with 1% lidocaine and epinephrine (1:100,000) was administered subcutaneously to facilitate hydrodissection, allowing separation of the skin from the underlying muscle. A no. 15 scalpel was used to make the incision from the medial canthus to the peak point and further to the lateral boundary. Following skin excision, strip resection of the preseptal orbicularis oculi muscle was performed. The exposed septum was tightened using bipolar cautery. In patients with superior orbital sulcus hollowness, orbicularis oculi muscle resection was not performed.

**Fig. 1:**
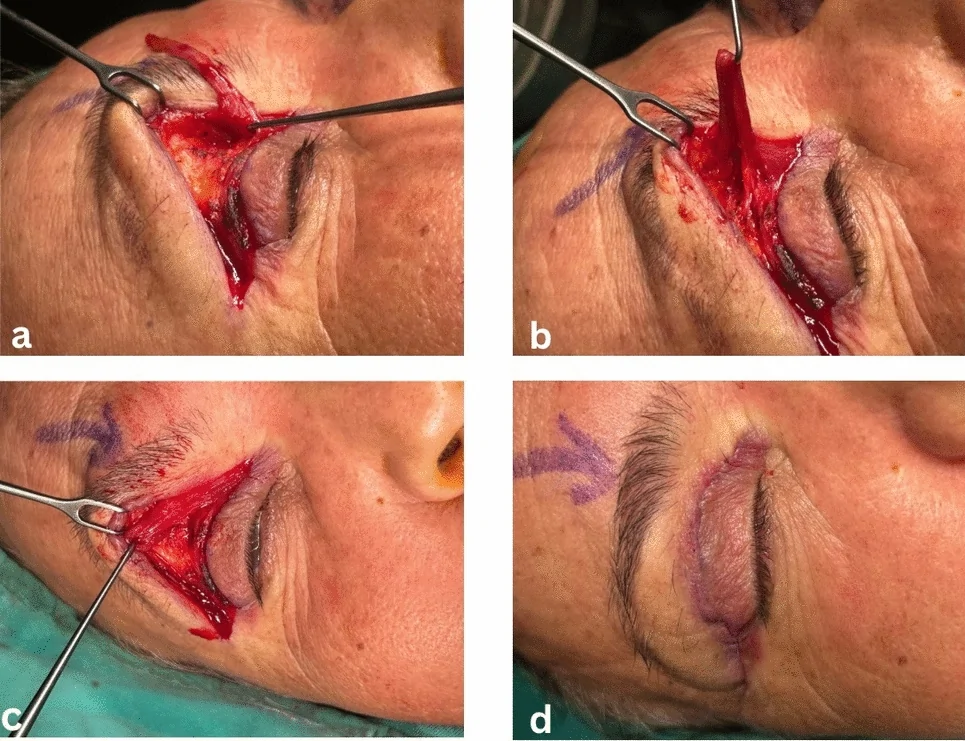
Intraoperative steps of the preseptal orbicularis oculi muscle flap in patient no 40. **a** After upper blepharoplasty skin excision, a medial-pedicled orbicularis oculi muscle flap is designed and elevated. **b** A subperiosteal pocket is created at the site of superior orbital sulcus hollowness. **c** The flap is folded and inset into the prepared pocket to restore medial sulcus volume. **d** Final wound closure with subcuticular sutures demonstrating tension-free skin adaptation

**Fig. 2:**
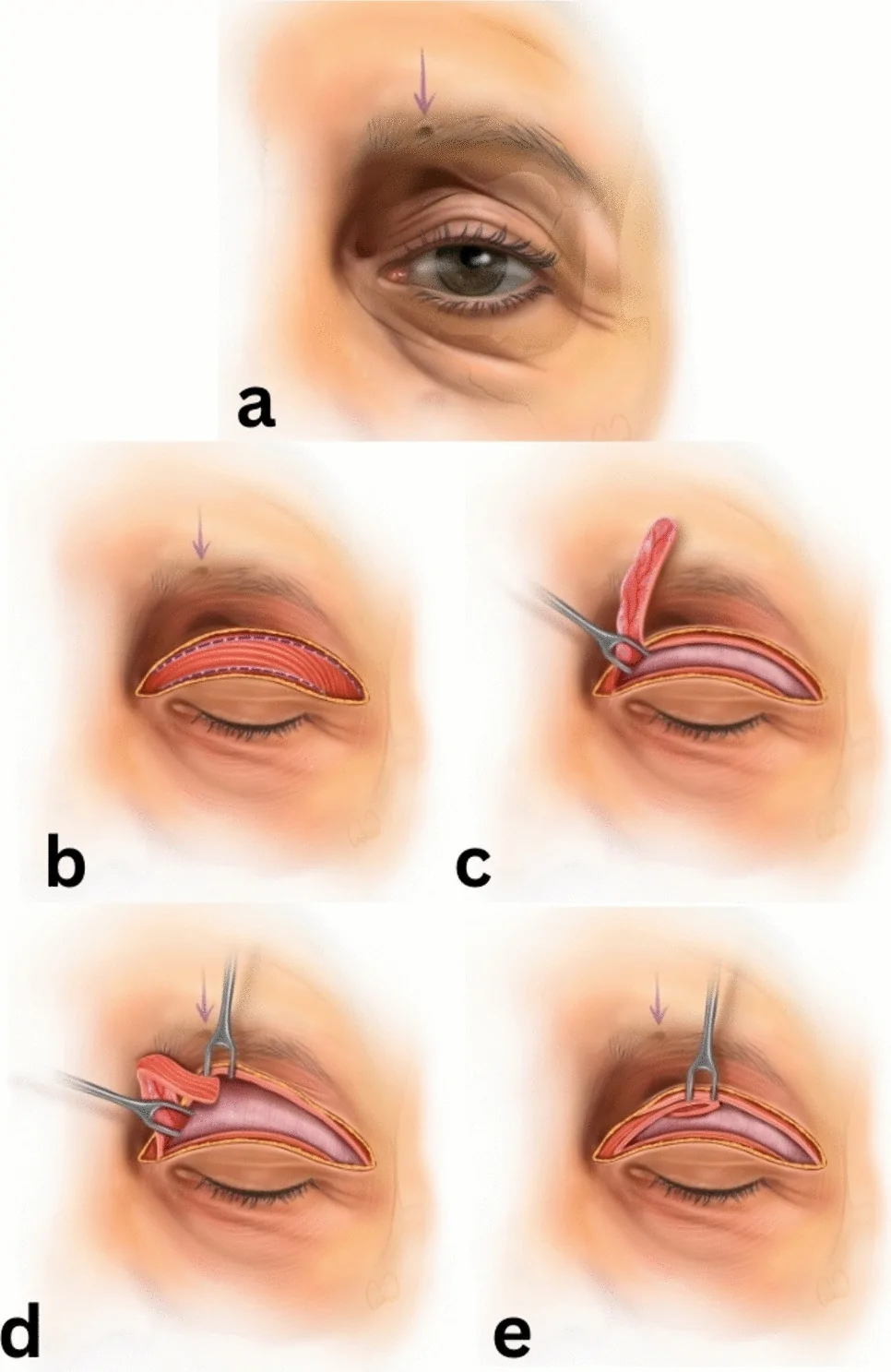
Illustration of the surgical steps for correction of superior orbital sulcus hollowness using a medial-pedicled preseptal orbicularis oculi muscle flap. **a** Preoperative appearance demonstrating marked superior orbital sulcus hollowness. **b** Skin excision and exposure of the preseptal orbicularis oculi muscle. **c** Elevation of a medial-pedicled flap from the preseptal orbicularis oculi muscle. **d** Advancement and folding of the muscle flap toward the superior orbital sulcus defect. **e** Final inset of the flap into the created pocket beneath the superior orbital sulcus

Depending on the vertical length of the upper eyelid, a 4–6 mm wide muscle flap with a medial pedicle was elevated, together with the septum, from the base to the pre-marked area. The caudal incision of the flap was extended 2–4 mm longer medially than the cranial incision. Using a gentle hook, the pedicle of the flap was carefully elevated, and a suitable pocket was created beneath the area responsible for the hollowness. Herniated fat tissues encountered during the procedure were reduced using electrocautery. A 6-0 round Vicryl suture was placed cranio-caudally through the periosteum at the pocket’s depth. The elevated flap was then folded upon itself, with the lateral edge adapted into the pocket. The remaining free portion of the folded flap was pulled laterally, aligned beneath the eyebrow area, and sutured in place. The skin was closed with 6-0 Prolene in a subcuticular manner (Figs. 1, 2). To prevent the incision from opening at the area of greatest tension, at the lateral canthus level, the skin was additionally closed with 6-0 Vicryl. OMNİSTRİPS® were applied over the incision lines. Following cold application, patients were discharged four hours postoperatively.

## Results

**Fig. 3:**
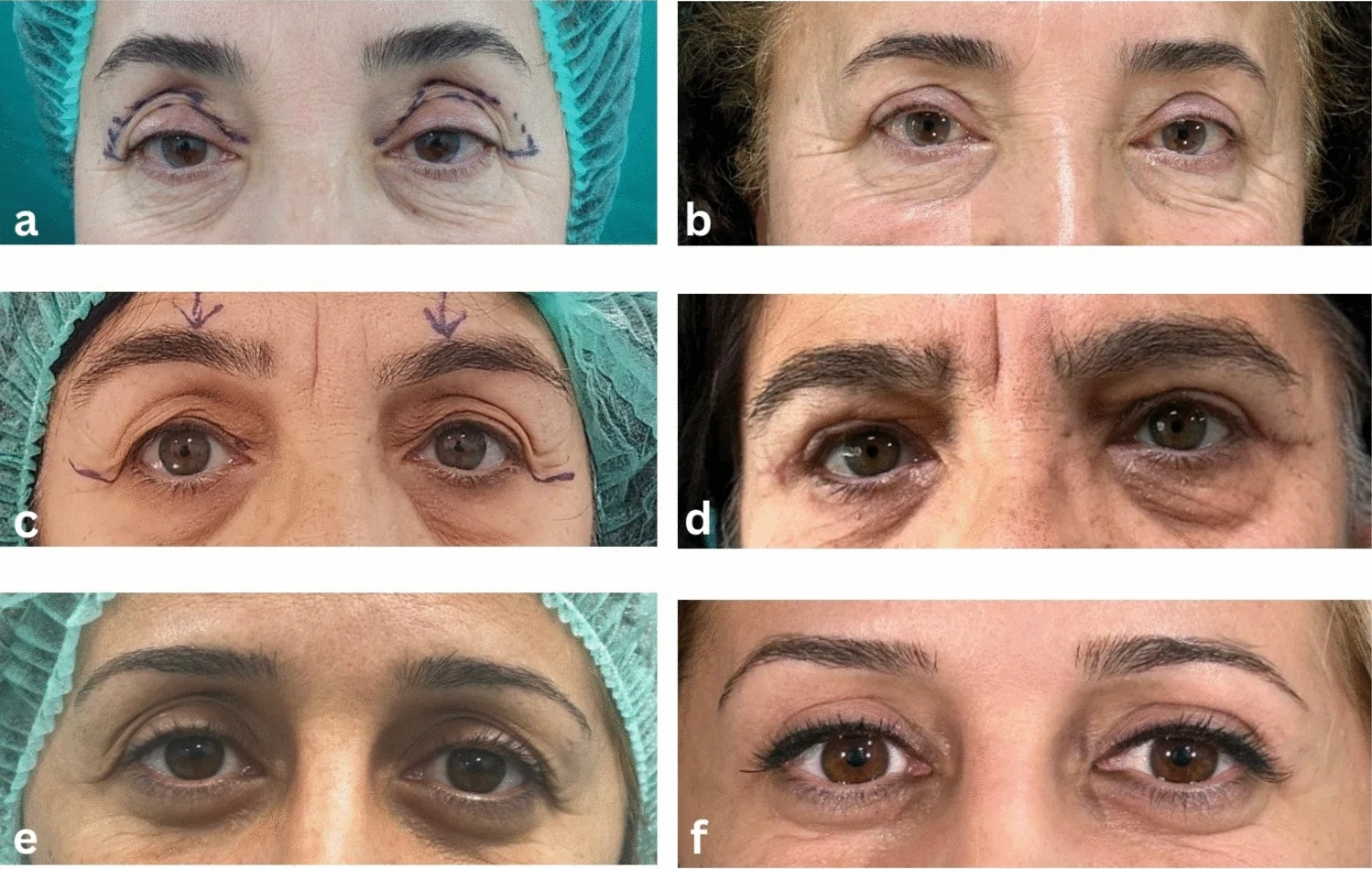
Representative preoperative and postoperative outcomes of patients undergoing upper blepharoplasty with POOM flap for superior orbital sulcus hollowness. **a**, **b** 54-year-old female (patient no. 12), preoperative and 1-year postoperative views. **c**, **d** 68-year-old female (patient no. 34), preoperative and 6-month postoperative views. **e**, **f** 44-year-old female (patient no. 43), preoperative and 2-year postoperative views

A total of 481 upper blepharoplasty procedures were performed during the study period. The mean follow-up duration was 8 months, and the mean age of the patients was 51.4 years. Among these, 45 patients underwent correction of superior orbital sulcus hollowness using a POOM flap. The most common early postoperative complications in both groups were transient lagophthalmos and edema, which resolved within 6 hours after surgery. No cases of infection, hematoma, or edema lasting longer than two weeks were observed in either group (Fig. 3).

In the standard blepharoplasty group, the most frequent late postoperative complication was prominent scar tissue in the medial canthal region (16 of 436 patients). Of these, 4 patients underwent revision with single Z-plasty, while the remaining patients experienced gradual improvement of their complaints over time. No cases of medial canthal scar formation were observed in the group treated with the POOM flap. In the group treated with the POOM flap, the most frequent late complication was numbness along the course of the supraorbital nerve. Patients with numbness received vitamin B supplementation. The numbness persisted for an average of 3.2 months but was not permanent in any case. There were no signs of flap necrosis in any patient during follow-up. In both groups, patients who reported preoperative complaints of ocular dryness were managed with lubricating eye drops for three weeks postoperatively.

All statistical analyses were performed using SPSS version 26. The primary outcome was the FACE-Q Adverse Effects score, transformed to a 0–100 scale according to the Rasch conversion table. The normality of FACE-Q score distributions was assessed using the Shapiro–Wilk test. Since the distributions in both groups deviated from normality (*p *< 0.001), the nonparametric Mann–Whitney U test was used to compare FACE-Q scores between groups. A *p* value less than 0.05 was considered statistically significant. FACE-Q scores for Group 1 (*n *= 436) and Group 2 (*n *= 45) are summarized in Table 1. The Mann–Whitney U test showed no statistically significant difference in FACE-Q Adverse Effects scores between the two groups (Mann–Whitney U = 8828.0, *p *= 0.251).

**Table 1:** FACE-Q adverse effects scores of study groups

	Group 1 (*n *= 436) conventional upper Blepharoplasty	Group 2 (*n* = 45) Blepharoplasty + POOM flap	*p* value/test
Mean ± SD	20.15 ± 10.21	20.04 ± 12.65	
Median (IQR)	19.0 (13–28)	24.0 (10–28)	
Minimum–Maximum	0–59	0–45	
Normality (Shapiro–Wilk)	*p *< 0.001	*p *< 0.001	
Statistical test			Mann–Whitney U = 8828.0
*p*-value			0.251

Values are presented as mean ± standard deviation (SD), median with interquartile range (IQR), and range. FACE-Q scores were transformed to a 0–100 scale using the Rasch conversion table. Normality was assessed with the Shapiro–Wilk test. Group comparisons were performed using the Mann–Whitney U test

## Discussion

Superior orbital sulcus hollowness arises from tissue retraction, often due to structural changes, trauma, or surgical interventions such as excessive fat removal during upper blepharoplasty. This results in volume loss in the medial half of the superior orbital sulcus and manifests as a tired appearance.6 It can also develop secondary to age-related changes in orbital anatomy and displacement of intraorbital soft tissues [5, 9]. If left unaddressed, this deformity may become more pronounced following blepharoplasty. Interventions for superior orbital sulcus hollowness have often focused on fat redistribution or autologous fat grafting [7, 10]. However, fat tissue vascularization may be variable, and atrophy can occur following periorbital trauma [11]. Moreover, injecting fat grafts into the orbit carries inherent risks due to anastomoses with the internal carotid system, where fat embolism in the central retinal artery or other intracranial arteries may result in devastating consequences [12]. Nevertheless, when performed with appropriate technique and anatomical awareness, fat grafting can be a safe and effective option in facial applications, largely owing to the rich vascular supply of the region [13].

Among facial rejuvenation procedures, upper eyelid blepharoplasty is considered to have one of the lowest complication rates [14]. This is attributed to the upper eyelid’s alignment with gravity vectors and its rich vascular network [15]. Upper blepharoplasty techniques vary and may include skin-only excision or combined skin and orbicularis oculi muscle resection, depending on the surgical indication and surgeon preference. When muscle excision is performed, studies have shown that no significant functional impairment occurs as long as the excised amount does not exceed 4–5 mm [16, 17]. In our series, the most common early complication was transient lagophthalmos, caused by local anesthetic infiltration disrupting orbicularis closure dynamics. Performing the procedure under general anesthesia could avoid this issue, but for an average 35-minute operation, it may not be cost-effective.

In the late postoperative period of group 2, hypoesthesia along the supraorbital nerve tract was observed in 71% of patients. This occurred due to suturing the distal end of the flap anterior to the supraorbital notch. The sensory deficit was temporary, resolving within a few months as the 6-0 Vicryl sutures dissolved. No cases of flap necrosis were noted, supporting the reliability of the flap’s vascular supply.

For eyelid reconstruction, elevating the orbicularis oculi muscle as a flap has long been considered a successful approach. Circulation patterns of bipedicle Tripier flaps or medially pedicled flaps were described years ago [18]. The use of a medial-based orbicularis oculi muscle flap for contour correction has also been reported [19]. However, folding this flap to address medial orbital rim soft tissue deficiency responsible for superior orbital sulcus hollowness represents a new concept. Assessing flap circulation is crucial: after elevating the medial-pedicled orbicularis oculi muscle flap, waiting a few minutes allows evaluation of its viability. In our study, no clinical signs of necrosis were observed postoperatively.

Patients with a prior upper eyelid blepharoplasty were excluded from this study, since secondary cases often lacked reliable surgical documentation. Elevating a 3–4 mm wide muscle block as a flap from an eyelid with prior muscle excision could impair eyelid function. Therefore, in patients with uncertain surgical history, elevating a medial-pedicled orbicularis oculi muscle flap should be avoided. Superior orbital sulcus hollowness should be regarded as a pre-existing condition that becomes more apparent if not addressed during blepharoplasty [20].

The amount of soft tissue required to eliminate superior orbital sulcus hollowness varies among patients. However, the volume of the elevated medial-pedicled muscle flap is relatively uniform: approximately 4 mm thick, covering a 120° arc of the palpebral orbicularis. This explains why the deformity was completely corrected in some patients, while only partially improved in others. The main advantage of this technique lies in achieving patient-centered outcomes without significantly prolonging surgical time or causing additional complications, making it easily adoptable with a gentle learning curve.

Among candidates for blepharoplasty, dermatochalasis is the predominant presenting complaint; in the subset with superior orbital sulcus hollowness, patients frequently identify sulcus deficit as a principal contributor to perceived facial aging. Although precise quantification of patient-reported benefit is limited, postoperative FACE-Q scores in this cohort allowed comparison between conventional blepharoplasty and the adjunctive flap procedure, and did not indicate a deterioration in outcomes attributable to the adjunct technique.

The purpose of the POOM flap described herein is volume replacement in patients with pre-existing superior orbital sulcus hollowness. Evidence for flap viability in our series is limited to intraoperative assessment of perfusion prior to inset and postoperative clinical surveillance for signs of flap necrosis. Although no clinically significant volume loss was observed during the 6-month follow-up period, objective volumetric assessment was not performed, and subtle changes related to muscle atrophy or scarring cannot be excluded.

Quantitative methods to directly measure the flap’s functional performance remain limited. The FACE-Q Upper Blepharoplasty Adverse Effects panel represents one of the most informative instruments for evaluating the clinical success of this flap. Using this instrument to compare patients with superior orbital sulcus hollowness who underwent blepharoplasty with a POOM flap versus patients without hollowness who underwent standard blepharoplasty provides a clear method to delineate the flap’s functional contribution and its potential complication profile.

The cohort treated with the POOM flap was relatively small, which may reduce statistical power. The study was not designed to include a comparison group treated with alternative volume-restoration techniques for superior orbital sulcus hollowness; accordingly, our findings do not permit definitive conclusions from direct comparative evaluation regarding relative efficacy or safety. Finally, patient-reported outcomes were captured only with the FACE-Q Upper Blepharoplasty Adverse Effects panel, and the absence of additional, more objective measures may limit sensitivity to certain outcome domains.

## Conclusion

The POOM flap offers a safe and reproducible option for volume replacement in patients with superior orbital sulcus hollowness undergoing upper blepharoplasty. In our series, no flap necrosis was observed, and the procedure did not result in additional complications beyond those seen with standard blepharoplasty, as confirmed with the FACE-Q Adverse Effects panel. However, several limitations should be considered: The flap provides a relatively uniform volume that may not fully correct the deformity in all patients, transient sensory changes along the supraorbital nerve were observed, quantitative methods to objectively assess flap viability were lacking, and the number of patients treated with this technique was relatively small, reducing the generalizability of our findings. Despite these drawbacks, the technique represents a promising, anatomically sound alternative to fat grafting and other volume-restoration methods, with the potential to enhance aesthetic outcomes and patient satisfaction in carefully selected cases.

## Supplementary Information

Below is the link to the electronic supplementary material.

Supplementary file 1 (MP4)

Supplementary file 2 (MP4)

Supplementary file 3 (MP4)

Supplementary file 4 (MP4)
